# Nitrogen isotopic signatures and fluxes of N_2_O in response to land-use change on naturally occurring saline–alkaline soil

**DOI:** 10.1038/s41598-020-78149-w

**Published:** 2020-12-04

**Authors:** Arbindra Timilsina, Wenxu Dong, Jiafa Luo, Stuart Lindsey, Yuying Wang, Chunsheng Hu

**Affiliations:** 1 Key Laboratory of Agricultural Water Resources, Hebei Key Laboratory of Soil Ecology, Center for Agricultural Resources Research, Institute of Genetics and Developmental Biology, Chinese Academy of Sciences, Shijiazhuang, 050021 China; 2 University of Chinese Academy of Sciences, Beijing, 100049 China; 3 Land and Environment, AgResearch, Hamilton, 3240 New Zealand

**Keywords:** Ecology, Ecology, Environmental sciences

## Abstract

The conversion of natural grassland to semi-natural or artificial ecosystems is a large-scale land-use change (LUC) commonly occurring to saline–alkaline land. Conversion of natural to artificial ecosystems, with addition of anthropogenic nitrogen (N) fertilizer, influences N availability in the soil that may result in higher N_2_O emission along with depletion of ^15^N, while converting from natural to semi-natural the influence may be small. So, this study assesses the impact of LUC on N_2_O emission and ^15^N in N_2_O emitted from naturally occurring saline–alkaline soil when changing from natural grassland *(Phragmites australis)* to semi-natural [*Tamarix chinensis* (Tamarix)] and to cropland (*Gossypium* spp.). The grassland and Tamarix ecosystems were not subject to any management practice, while the cropland received fertilizer and irrigation. Overall, median N_2_O flux was significantly different among the ecosystems with the highest from the cropland (25.3 N_2_O-N µg m^−2^ h^−1^), intermediate (8.2 N_2_O-N µg m^−2^ h^−1^) from the Tamarix and the lowest (4.0 N_2_O-N µg m^−2^ h^−1^) from the grassland ecosystem. The ^15^N isotopic signatures in N_2_O emitted from the soil were also significantly affected by the LUC with more depleted from cropland (− 25.3 ‰) and less depleted from grassland (− 0.18 ‰). Our results suggested that the conversion of native saline–alkaline grassland with low N to Tamarix or cropland is likely to result in increased soil N_2_O emission and also contributes significantly to the depletion of the ^15^N in atmospheric N_2_O, and the contribution of anthropogenic N addition was found more significant than any other processes.

## Introduction

Nitrous oxide (N_2_O) is a major long-lived anthropogenic greenhouse gas with about 265–298 fold greater potential for global warming in the atmosphere compared to carbon dioxide^1^. It is also an ozone-depleting substance^2^, produced mainly in the soil from nitrification and denitrification processes^3^. Its concentration in the atmosphere has increased to 331 ppb^4^ from 270 ppb in the pre-industrial age^5^. This increase of N_2_O in the atmosphere is mainly attributable to rise in anthropogenic nitrogen (N) input to soil^6,7^ and this anthropogenic N input to soil increases as more natural ecosystems are converted to croplands.

Soil salinity can influence N_2_O flux in different ways. An increase in salinity in a non-saline soil can increase^8^ or have no effect on N_2_O emission^9^. Similarly, on naturally occurring saline soils, both decreases^8^ and increases^10^ in the N_2_O flux have been found in response to increase in the salinity. These results suggest an ambiguous role of salinity in N_2_O emission. Some meta-analyses^11,12^ have reported that alkaline soil emits less N_2_O than neutral or acidic soil. In alkaline soil NH_4_ may be converted to NH_3_ and volatilize to the atmosphere whereas NH_4_ is retained in acid soil, favoring N_2_O formation^13^. N loss from alkaline soil may be high in total, but if much of the N is lost in the form of NH_3_ there may be less NH_4_ available for nitrification and subsequent denitrification. This evidence suggests that in naturally occurring saline–alkaline soil, the influence of both salinity and alkalinity may significantly affect the N_2_O formation processes. So, quantifying N_2_O flux from the saline–alkaline soil may help to increase knowledge on its contribution to soil-atmosphere exchange of N_2_O.

Land-use change (LUC) from natural to semi-natural or artificial ecosystems can have different effects on N_2_O emission^14–16^. Specifically, conversion from natural to artificial ecosystems with the addition of N fertilizer significantly increases N_2_O emission while conversion to semi-natural may or may not increase the emission^16–18^. LUC directly impacts on soil physical, chemical and biological properties^19,20^, the main factors affecting N_2_O emission^21,22^. N_2_O emission from soil is reduced when pasture is forested^14^, while conversion of rainforest to pasture or plantation leads to an increase in N_2_O emission^15^. A recent study found that the conversion of a conventional agricultural field to bio-energy crops had no effect on N_2_O emission^23^. Therefore, knowing which LUC practice is appropriate in terms of lower N_2_O emissions, and its implementation could mitigate N_2_O emission to the atmosphere and associated impact of climate change. Moreover, various LUC practices^14,15,23^ have different or no effect on N_2_O emission, indicating that LUC is rather an indirect cause of N_2_O emission. The main reason for the differences in N_2_O emission due to LUC is probably the alteration of the controlling factors of N_2_O production and reduction processes in the soil. So, quantifying N_2_O flux from LUC, along with soil physical and chemical parameters, would further enable understanding of the main driving factors for N_2_O production and consumption in the soil.

N has two stable isotopes i.e., ^14^N and ^15^N. δ ^15^N of a sample is the deviation of the samples’ ^15^N/^14^N from the respective isotope ratio of the reference material^24^. Previously, the ^15^N in N_2_O emitted from soil has been used to identify the processes for N_2_O production i.e. nitrification and denitrification; however, using only ^15^N values in N_2_O may mislead the interpretation^7^ as both the processes generally occur in the soils, possibly in different horizons or niche. The ^15^N in N_2_O emitted from soil depends on the ^15^N content of the substrates i.e. NH_4_ and NO_3_, different microbial community composition, pH, temperature and substrate availability^24,25^. Although it is difficult to predict the sources of N_2_O emission using solely ^15^N signatures in the N_2_O, these values could be used to distinguish between N_2_O emitted from natural and artificial ecosystems^25^. N addition in the artificial ecosystems increases the N availability which depletes the ^15^N in N_2_O^25^. So, LUC from natural ecosystems to cropland may not only influence the N_2_O fluxes but also the ^15^N in the emitted N_2_O as N availability is altered. For example, mean ^15^N in N_2_O emitted from natural tropical forest, sub-tropical forest and subarctic soil are − 18.0‰, − 14.3‰, and − 13.0‰, respectively ^25–27^; while more depleted after N application i.e., − 37.9‰^28^ to − 34.3‰^25^ in fertilized soil. The difference of ^15^N in N_2_O is useful to distinguish N_2_O emitted between fertilized and natural soils, and it arises from anthropogenic N addition to soil^25,28^. Moreover, application of N fertilizer leads to high concentrations of NO_3_ in the soil, resulting in a decrease in N_2_O reduction to N_2_ and therefore a higher N_2_O to N_2_ ratio from the denitrification process^29^. The reduction of N_2_O to N_2_ through denitrification leads to 1–24‰ ^15^N enrichment of the remaining N_2_O^30^. So, differences in the capacity to reduce N_2_O to N_2_ between various ecosystems may also influence the ^15^N in emitted N_2_O.

To feed the world’s growing population requires an additional 2.7–4.9 Mha of cropland per year on average^31^. Due to limited land resources, natural saline–alkaline areas are being reclaimed for producing food^32^. Agricultural soil alone will contribute about 59% of total global N_2_O emissions by 2030^3^ as fertilizer application will need to increase by about 35–60%^33^. Therefore, it is important to quantify, and develop measures to mitigate increases in N_2_O fluxes resulting from the conversion of natural saline–alkaline grassland to cropland. Furthermore, *Tamarix chinensis* (Tamarix), a salt tolerant native species of shrub, is commonly used for the restoration of saline–alkaline soil in coastal areas in China (semi-natural ecosystem)^34^. Local governments have launched a coastal ecological restoration program promoting the planting of Tamarix^35^; however, its effect on N_2_O emission is unknown. Though Zhang et al.^36^ reported the differences in N_2_O emission from various natural vegetation in saline–alkaline coastal areas, the impact of LUC from natural to semi-natural or artificial ecosystems on the dynamics of N_2_O emissions from saline–alkaline soil is unknown. Moreover, different plant species have been reported to modify the soil characteristics in varying ways, resulting in significant changes in N_2_O fluxes^37^. Therefore, we hypothesize that: (1) LUC from native saline–alkaline ecosystem (grassland) to semi-natural (Tamarix) may significantly influence N_2_O flux, along with soil environmental variables (soil temperature, soil moisture, ammonium, nitrate), because of the difference in plant species but have no effect on the ^15^N in N_2_O emitted from the soil because there is no addition of anthropogenic N and (2) LUC from native saline–alkaline ecosystem (grassland) to artificial (cropland) may influence both N_2_O flux and the^15^N in N_2_O due to anthropogenic N addition and changes in management practices. Therefore, we expect that the ^15^N in emitted N_2_O could be used to distinguish N_2_O emitted between unfertilized (natural and semi-natural ecosystems) and fertilized (cropland) ecosystems but not between different unfertilized ecosystems (grassland and Tamarix).

## Methods

### Site description

**Figure 1:**
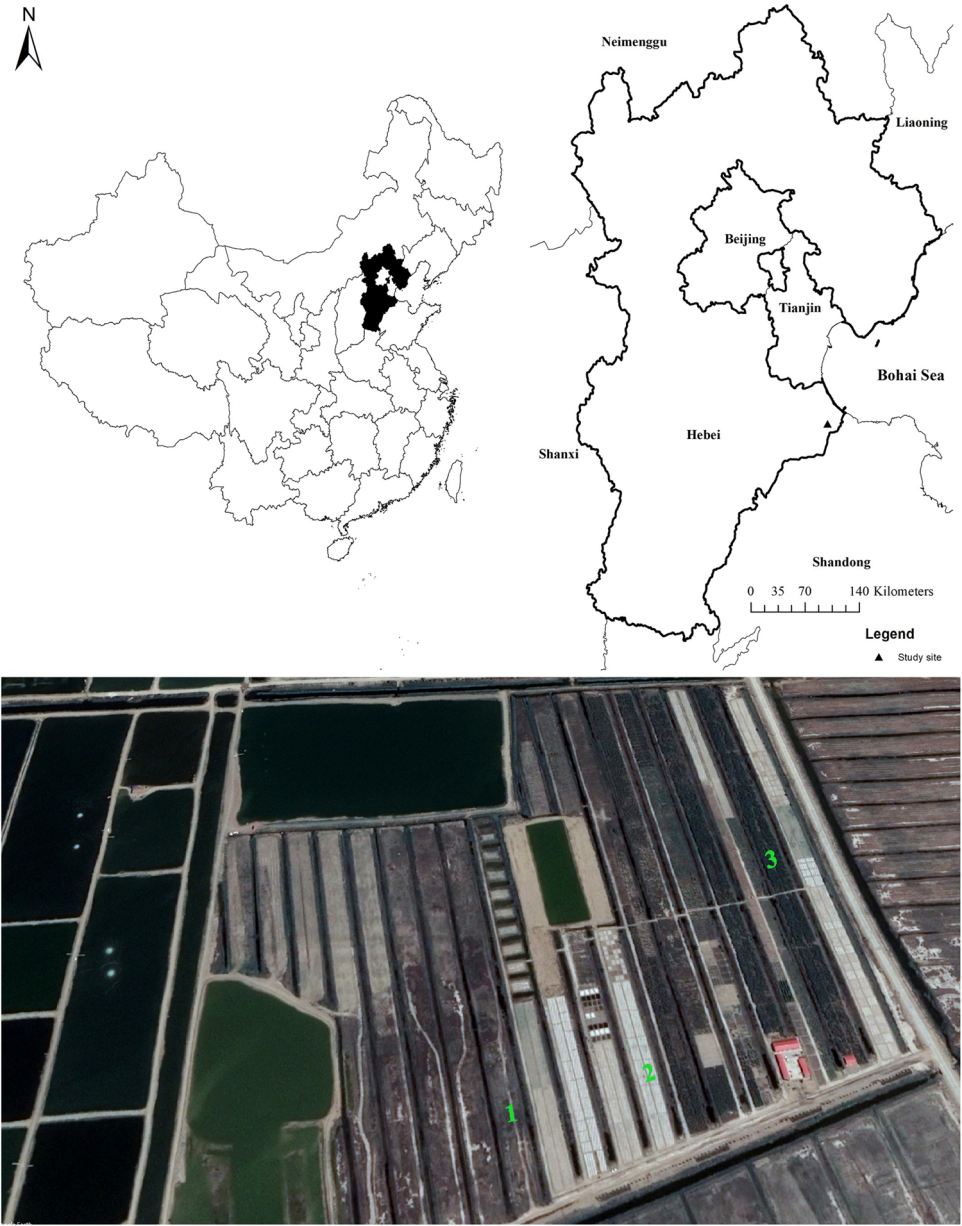
Map and an aerial photo of the study site. 1, 2 and 3 in the aerial photo represent the positions of the grassland, cropland, and Tamarix, respectively. The map was created using ArcGIS (v10.3.1) (ESRI Inc.) and Google Earth.

The study was carried out from April 2017 to June 2018 at the Haixing experimental station of the Center for Agricultural Resources Research (CARR), Institute of Genetics and Developmental Biology (IGDB), Chinese Academy of Sciences (CAS). This site is located near the Bohai sea in Haixing county (117°33′5″ E, 38°09′59″N) of Hebei province, China (Fig. 1). The site has a semi-humid monsoon climate with more than 75% of precipitation occurring during the rainy season, i.e. from July to September. The mean annual precipitation is 582 mm. The groundwater table is at 0.9–1.5 m depth. The soil in this area is classified as solonchak (18.1% clay and 7.8% sand). The salt content in the area ranges from 3 to 20 g kg^−1^ soil^38^.

In 2008, the native grassland was converted to Tamarix and cropland with the aim of reclamation of the saline–alkaline soil. The Tamarix stand was left to grow naturally after plantation. For this reason, we consider it as a semi-natural ecosystem. The cropland (artificial ecosystem) has permanent plots 7.25 m × 7.25 m in size, which were left fallow after conversion until 2014. During the fallow period, the cropland plots were irrigated (180 mm per year) around early January with saline groundwater. The irrigated water freezes from January to late February or early March as air temperatures are mostly below 0 °C. The salinity of the irrigated groundwater was 7–27 g l^−1^ ^38^. This practice of irrigation reduces the salinity in the soil and decrease the salt stress on subsequently planted cotton seedlings^38^. Since 2014, during each March, the cropland has been covered with plastic film until the sowing of the cotton to reduce the evapotranspiration^38^. The cropland received 400 kg N ha^−1^ year^−1^ applied during May every year (200 kg N ha^−1^ organic fertilizer + 200 kg N ha^−1^ diammonium phosphate) before sowing cotton since 2014. During this experimental period, cropland was fertilized on 7th May 2017 and 6th May 2018 and irrigation occurred on 10th Jan 2018. The irrigated water had melted completely by 21st Feb 2018. Other details of the three ecosystems are reported in Table 1.

**Table 1:** Management practices, dominant vegetation and some physical and chemical soil parameters of the three ecosystems.

S. no	Ecosystem	Management practice	Dominant plant species	Soil bulk density (g cm^−3^)	Soil pH	Soil salinity (mS cm^−1^)
1	Grassland	Native grassland, no grazing, no cutting, no fertilizer	Common reed *(Phragmites australis*)	1.56 ± 0.04a	8.74 ± 0.07	2.29 ± 0.19
2	Cropland	Converted from the grassland, fertilizer use (organic + chemical), irrigation once a year (saline water)	Cotton (*Gossypium* spp., Lumian 28)	1.58 ± 0.01a	8.45 ± 0.06	2.17 ± 0.29
3	Tamarix	Converted from the grassland, no fertilizer use, no cutting, no litter removal	Tamarix (*Tamarix chinensis*)	1.38 ± 0.02b	8.58 ± 0.04	2.15 ± 0.10

Different letters in the row indicate significant differences (< 0.05), while no letters means no difference.

### Gas sampling

In each ecosystem, four closed static chambers were randomly placed. The chambers were made of polyvinyl chloride (PVC) and measured 60 × 20 × 40 cm (L × B × H) and each chamber contained a fan to homogenize the air. The chambers were fitted with a thermometer and a sampling tube with a three-way stopcock. Both sampling tube and thermometer were sealed where they passed through the surface of the chamber to prevent leakage. Five 40-ml gas samples were taken for N_2_O concentration analysis at 20 min intervals using a glass syringe, while two 160-ml gas samples were taken at 0 and 80 min and stored in glass bottles for δ ^15^N-N_2_O analysis. Gas was sampled between 8:00 AM to 12:00 PM. Sampling was done twice to thrice in a month during March to September (warm season) while once in a month during October to February (cold season).

### N_2_O concentration measurement, flux calculation, and ^15^N isotope determination

The concentration of N_2_O was measured using gas chromatography (Agilent GC-6820, Agilent Technologies Inc., Santa Clara, CA, USA) equipped with ^63^Ni electron capture detection (ECD) in the laboratory of CARR, IGDB, CAS, Shijiazhuang, Hebei. The concentrations of N_2_O were calculated based on the measured peak areas relative to the peak areas measured from reference standards which were run twice before and after every fifteen gas samples.

The N_2_O flux was calculated using the following equation from Li et al.^39^.

1 $${\text{F}} = {\text{ M }} \times {\text{ V }} \times {\text{ A}}^{{ - {1}}} \times \, \Delta {\text{C}} \times \Delta {\text{t}}^{{ - {1}}} \times { 273 } \times \, \left( {{273 } + {\text{ T}}} \right)^{{ - {1}}} \times {\text{ P }} \times \, \left( {{\text{P}}^{0} } \right)^{{ - {1}}} \times { 6}0$$

where F is the N_2_O flux (μg N_2_O-N m^−2^ h^−1^), M is the molecular weight of N_2_O-N, V is the volume of the chamber (m^3^), A is the soil surface area occupied by the chamber base (m^2^), ΔC × Δt^−1^ is the slope of N_2_O accumulation in the chamber with the time change (10^–6^ min^−1^), T is the air temperature (°C) inside the chamber, P is the atmospheric pressure (hPa) on the sampling time and P^0^ is standard atmospheric pressure.

Annual cumulative emission rate was calculated by interpolating the N_2_O flux from four replicate chambers during measured days and the interval between sampling days. While calculating annual emission rate, it was assumed that there was no emission of N_2_O from 10 Jan to 21 Feb, 2018 in the cropland because of the frozen irrigated water on the surface (up to 18 cm thickness which was shrinking when the temperature rising). This assumption might underestimate the annual cumulative emissions. However, for grassland and Tamarix the rate for the whole year was calculated.

The gas samples (160 ml) were passed through a chemical trap [NaOH + Mg(ClO_4_)_2_] (FINNIGAN PRECON) to remove CO_2_ and H_2_O using a helium flow of 10–15 ml min^−1^. Using stainless steel trap, the gas sample was passed through liquid nitrogen. After this cryofocusing step, the gas sample passed into a GC (FINNIGAN GC). Finally, the δ ^15^N of the N_2_O was measured using an Isotope Ratio Mass Spectrometer (IRMS) (Delta V Plus. Thermo Fisher, Germany). δ ^15^N of data reported in this study are in unit of per mill (‰) relative to international standard (atmospheric N_2_). As the N_2_O in the sample represented the isotopic composition of both atmospheric and soil-emitted N_2_O, the following equation from Snider et al.^40^ was used to calculate the δ ^15^N of soil-emitted N_2_O.

2 $$\delta^{{{15}}} {\text{N of soil - emitted N}}_{{2}} {\text{O}} = (\delta^{{{15}}} {\text{N}}_{{\text{measured x}}} {\text{C N}}_{{2}} {\text{Omeasured}} - \delta^{{{15}}} {\text{N}}_{{\text{atmosphere x}}} {\text{C}}_{{{\text{atmosphere}}}} /\left( {{\text{C N}}_{{2}} {\text{O}}_{{{\text{measured}}}} - {\text{C N}}_{{2}} {\text{O}}_{{{\text{atmosphere}}}} } \right)$$

where δ ^15^N _measured_ and C N_2_O _measured_ are the δ ^15^N and concentration of the N_2_O sample at time 80 min after the closure of the chamber, while the δ ^15^N _atmosphere_ and C N_2_O _atmosphere_ are the δ ^15^N and concentration of the sample at time zero (immediately after the closure of the chamber). When the fluxes were lower than 10 N_2_O-N µg m^−2^ h^−1^, the ^15^N analyses were excluded from the results due to errors introduced with lower fluxes.

### Measurement of soil parameters

Soil temperature at 5 cm depth was taken using a thermometer inserted into the soil. Each day after the gas sample collection, soil samples (0–20 cm) were collected from the area nearby the chambers. Thermo-gravimetric technique (oven-drying) method was used to measure the soil moisture content. Water filled pore space (WFPS) was calculated using a formula as stated in Eq. (3):

3 $${\text{WFPS }}\left( \% \right) = \left( {\text{SWC x BD}} \right)/{1} - \left( {{\text{BD}}/{\text{PD}}} \right) \times {1}00\%$$

where SWC is soil water content (g g^−1^), BD is bulk density (Mg m^−3^), and PD is particle density (2.65 Mg m^−3^).

For soil pH and electrical conductivity (Ec), 10 g of air dried (< 2 mm) soil sample was weighed and mixed with 25 and 50 ml of deionized water, respectively. Then the mixture was mechanically shaken for 1 h. pH was determined in a suspension with a pH meter (METTLER TOLEDO FE20) at 1:2.5 soil–water ratio. Ec was measured using an Ec meter (METTLER TOLEDO SG7) with 1:5 soil–water ratio at room temperature. Soil ammonium (NH_4_-N) and nitrate (NO_3_-N) concentrations were measured using the KCl extraction method. For this, 10 g of fresh soil was mixed with 50 ml of freshly prepared 1 M KCl and the mixture was shaken for one hour, then it was filtered through Whatman 42 filter paper. Then, NH_4_-N and NO_3_-N concentrations of the filtrate were measured by using a Smartchem140 and a UV spectrophotometer, respectively.

### Statistics

Data were not normally distributed for all variables. Several possible transformations were tried without success. As our main objectives were to examine differences in N_2_O fluxes and ^15^N in soil emitted N_2_O in different ecosystems, we conducted the Kruskal Wallis ANOVA (analysis of variance) followed by the Mann Whitney test. The same analysis was used for other measured soil parameters. Similarly, differences in annual cumulative flux between ecosystems were computed through the Kruskal Wallis ANOVA followed by the Mann Whitney test. Spearman correlation analysis was applied to examine the relationships among the measured variables and N_2_O flux. When *p* values were less than 0.05 the differences was considered significant. All the figures and statistical analyses were computed in Origin Pro 8 (Origin Lab Ltd., Guangzhou, China).

## Results

### Soil environmental variables

**Figure 2:**
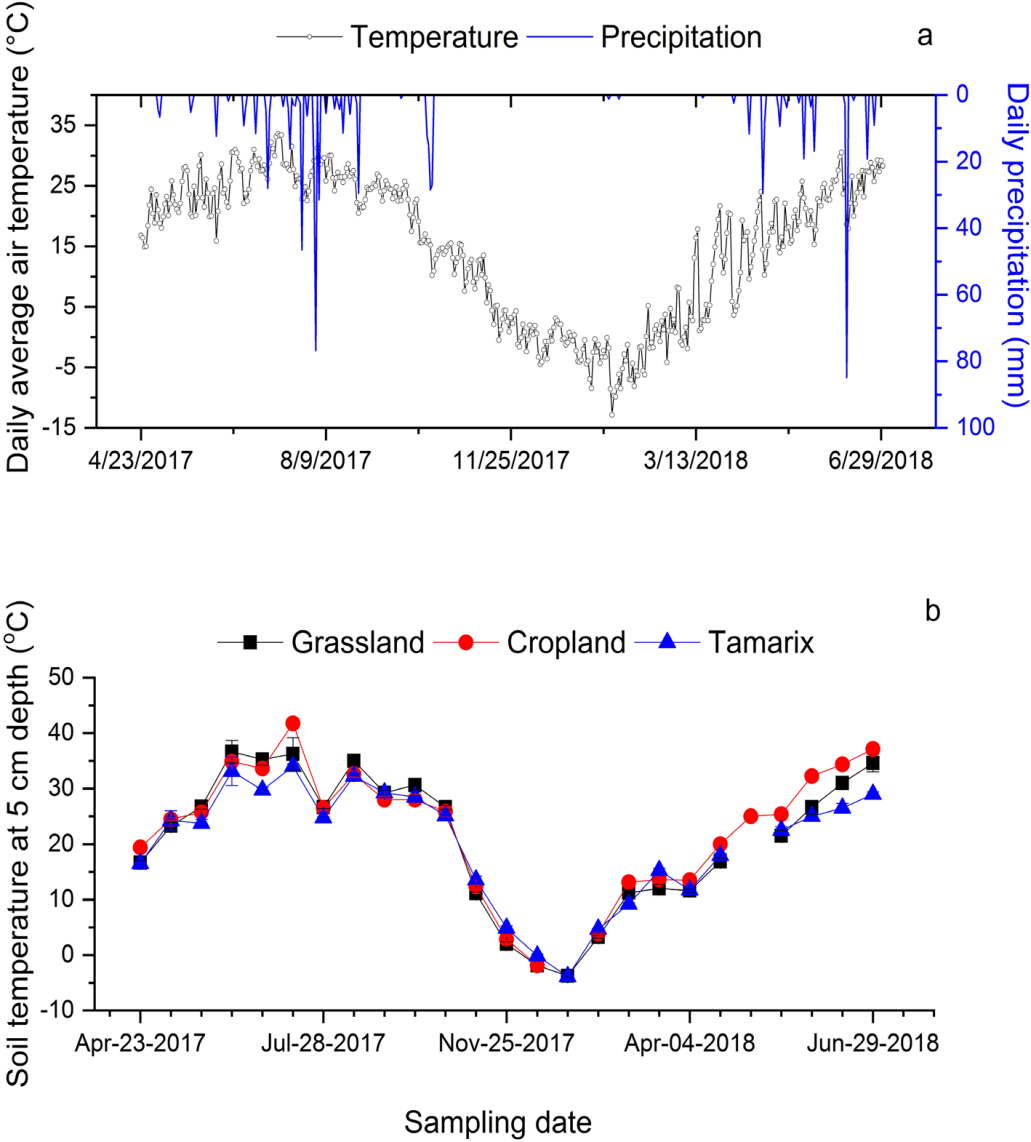
Daily average air temperature and precipitation at the study site during the study period (**a**) and soil temperature at 5 cm depth taken at the time of gas sampling (**b**). Error bars represent mean ± standard error (SE) (n = 4).

The pattern of soil temperature was consistent with the air temperature (Fig. 2a,b). Soil temperature at 5 cm soil depth showed a clear and similar seasonal variation (high in summer and low in winter) in all ecosystems. The lowest temperature was − 4 °C reported in January while the highest temperature was 42 °C in July. Soil temperature at 5 cm depth at grassland was similar to the cropland and Tamarix. While the Tamarix had significantly (p < 0.05) lower soil temperatures than the cropland. The median soil temperature was 24.5 °C, 25.3 °C and 23.5 °C in the grassland, cropland and Tamarix, respectively.

**Figure 3:**
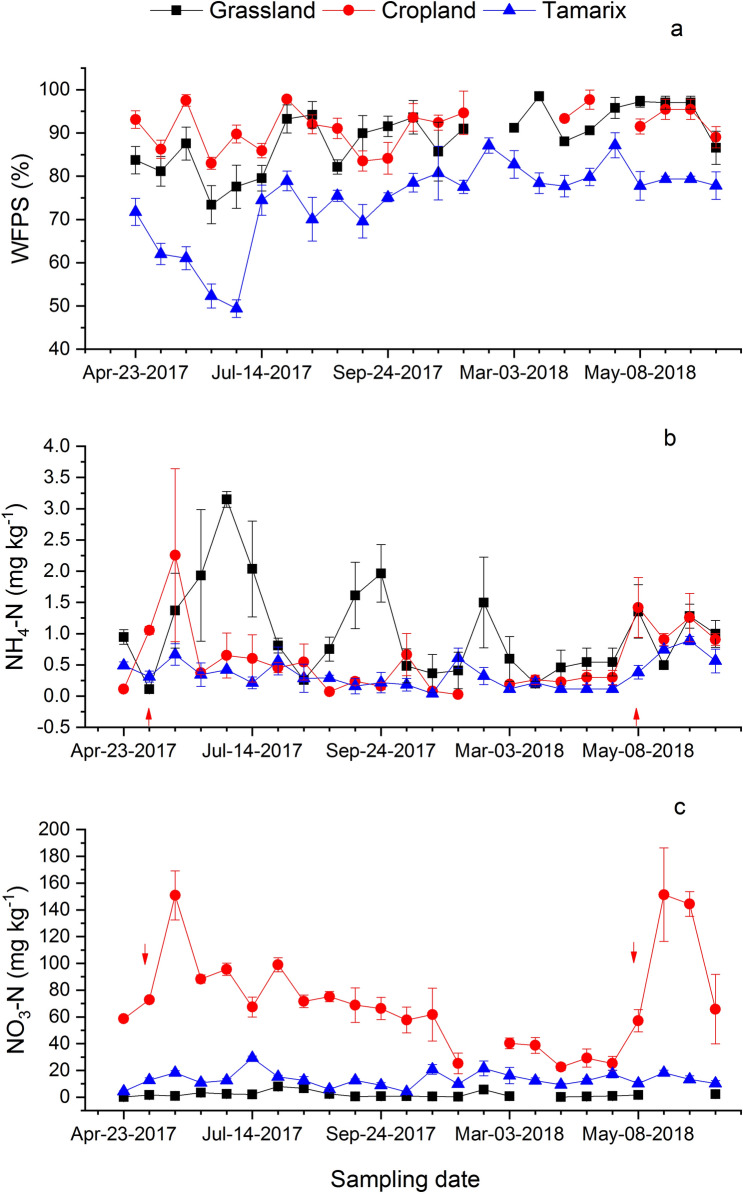
Soil water-filled pore space (WFPS) (**a**), Soil NH_4_ (**b**), and NO_3_ (**c**) of the top 20 cm soil. The arrows represent fertilizer application event. Each point represents athematic mean of n = 1–4 ± SE.

The overall WFPS of the Tamarix was significantly less (p < 0.001) than the grassland and cropland. The median value of WFPS in the grassland was 89.6% (ranging from 66.9 to 99.95%), cropland was 90.4% (ranging from 73.32 to 99.97%) and Tamarix was 76.2% (ranging from 44.4 to 97.0%). As water table was around 0.9–1.5 m, normally WFPS exceeded 70% in all ecosystems (Fig. 3a).

Soil NH_4_ was significantly (p < 0.01) higher in the grassland compared to the cropland and Tamarix. Overall, median NH_4_ concentration in the grassland was 0.55 mg kg^−1^ (ranging from 0.006 to 4.0 mg kg^−1^), 0.35 mg kg^−1^ (ranging 0.006–6.4 mg kg^−1^) in the cropland and 0.31 mg kg^−1^ (ranging from 0.01 to 1.2 mg kg^−1^) in the Tamarix. Grassland and cropland showed higher temporal variation in soil NH_4_ than the Tamarix during the sampling period (Fig. 3b). After fertilization of the cropland, there was a peak in NH_4_ content.

Soil NO_3_ was significantly different (p < 0.001) among all three ecosystems. The order of soil NO_3_ was: cropland > Tamarix > grassland. The median concentration of NO_3_ in the grassland was 1.0 mg kg^−1^ (ranging 0.004–14.0 mg kg^−1^), 65 mg kg^−1^ (6.4–209 mg kg^−1^) in the cropland and 12.3 mg kg^−1^ (ranging from 2.6 to 34.30 mg kg^−1^) in the Tamarix. For some sampling dates, NO_3_ was below the limit of detection in the grassland soil. Fertilizer application in the cropland led to a peak in NO_3_ content in the soil (Fig. 3c).

### N_2_O fluxes and annual cumulative emission

**Figure 4:**
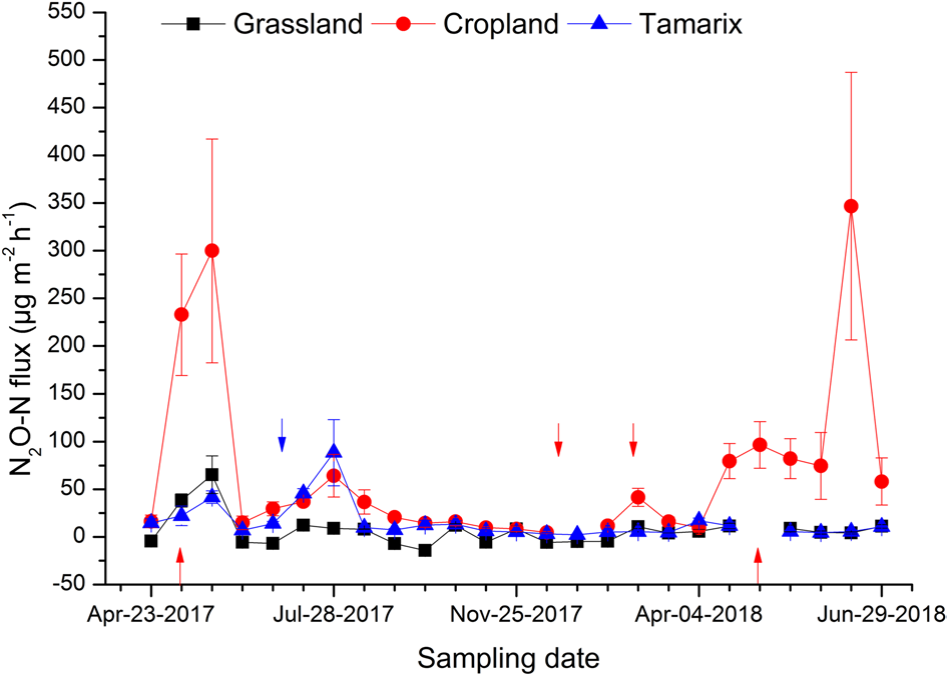
N_2_O flux from grassland, cropland, and Tamarix. Each point represents the arithmetic mean and standard error of four replicates. Arrows pointing upward indicate fertilization events on the cropland, while those pointing downward indicate the presence of dead pill-bugs in the Tamarix or irrigation and covered by the plastic film in the cropland (left to right). Red color arrows represent specific events in cropland and blue for Tamarix.

Among the 24 sampling occasions, 9 occasions were found negative fluxes in the grassland, but in the cropland and Tamarix there were always positive fluxes (Fig. 4). Overall, N_2_O fluxes were significantly different (p < 0.001) among the ecosystems. The median N_2_O flux was 4.0 N_2_O-N µg m^−2^ h^−1^ (ranging from − 22.0 to − 1.1 for negative flux and 2.8 to 117.7 4 N_2_O-N µg m^−2^ h^−1^ for the positive flux, over the study period), 25.3 N_2_O-N µg m^−2^ h^−1^ (ranging from 2.0 to 678.04 N_2_O-N µg m^−2^ h^−1^) and 8.2 N_2_O-N µg m^−2^ h^−1^ (ranging from 0.5 to 179.0 N_2_O-N µg m^−2^ h^−1^) from the grassland, cropland and Tamarix, respectively. The peak fluxes in the cropland occurred after the application of fertilizer (Fig. 4). In 2017, after fertilization the N_2_O peak lasted for two weeks. While in 2018, on the day of fertilization there was a small increase, then the highest peak occurred in the 4th week after fertilization. Results for February 2018 and the 3rd week after fertilization in 2018 are not reported because it was noted that there were unusually high concentrations of N_2_O (4 times higher than usual atmospheric concentration) in all samples taken at time zero, which may have led to errors in interpretation of results. For two of the sampling points, high N_2_O emissions from Tamarix were observed. This occurred during the decomposition of a large number of pill-bugs that had died at the site (the reason for the pill-bug deaths is unknown).

**Figure 5:**
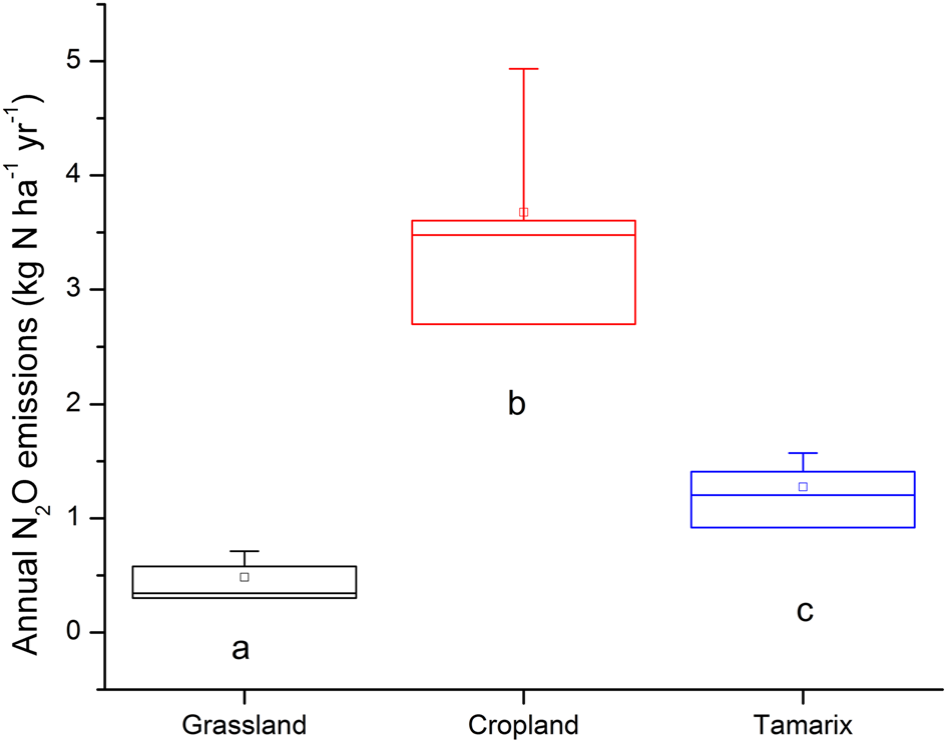
Box plot for annual N_2_O emissions (n = 4). Different letters indicate significant difference (p < 0.05) and square represents mean values.

The annual cumulative N_2_O emissions were significantly different (p < 0.05) among all three ecosystems. The annual cumulative N_2_O emissions increased in the order of cropland > Tamarix > grassland. Cropland emitted 3.5 kg N_2_O-N ha^−1^ year^−1^ (ranging from 2.7 to 3.9 kg N_2_O-N ha^−1^ year^−1^) about 1.7 times more than the Tamarix, which emitted 1.3 kg N_2_O-N ha^−1^ year^−1^ (ranging from 0.9 to 1.6 kg N_2_O-N ha^−1^ year^−1^), and 7 times more than the grassland (0.5 kg N_2_O-N ha^−1^ year^−1^, ranging from 0.3 to 0.7 kg N_2_O-N ha^−1^ year^−1^) (Fig. 5).

### Relationship between soil environmental variables and N_2_O flux

Spearman correlation analysis showed various relationships between N_2_O flux and soil environmental variables measured at three studied ecosystems (Table 2). In grassland, there was no significant relationship between N_2_O flux and any of the measured soil parameters. In the cropland, the analysis showed significant positive correlations of N_2_O flux with soil temperature, NH_4_ content, and NO_3_ content. There was no significant correlation between N_2_O emission and WFPS in the cropland. Analysis of the Tamarix results showed that there were significant positive correlations of N_2_O flux with soil temperature and NO_3_ content, while there was a negative relationship with WFPS.

**Table 2:** Spearman correlation analysis between soil environmental variables and N_2_O flux in different ecosystems.

Ecosystems/soil parameter	Grassland	Cropland	Tamarix
Soil temperature at 5 cm depth	0.08	0.426*	0.412*
WFPS	0.11	0.15	− 0.27*
NH_4_	− 0.1	0.50*	0.194
NO_3_	− 0.06	0.44*	0.28*

“*” represents significant relationship (p < 0.05).

### ^15^N isotopic signature of soil-emitted N_2_O

**Figure 6:**
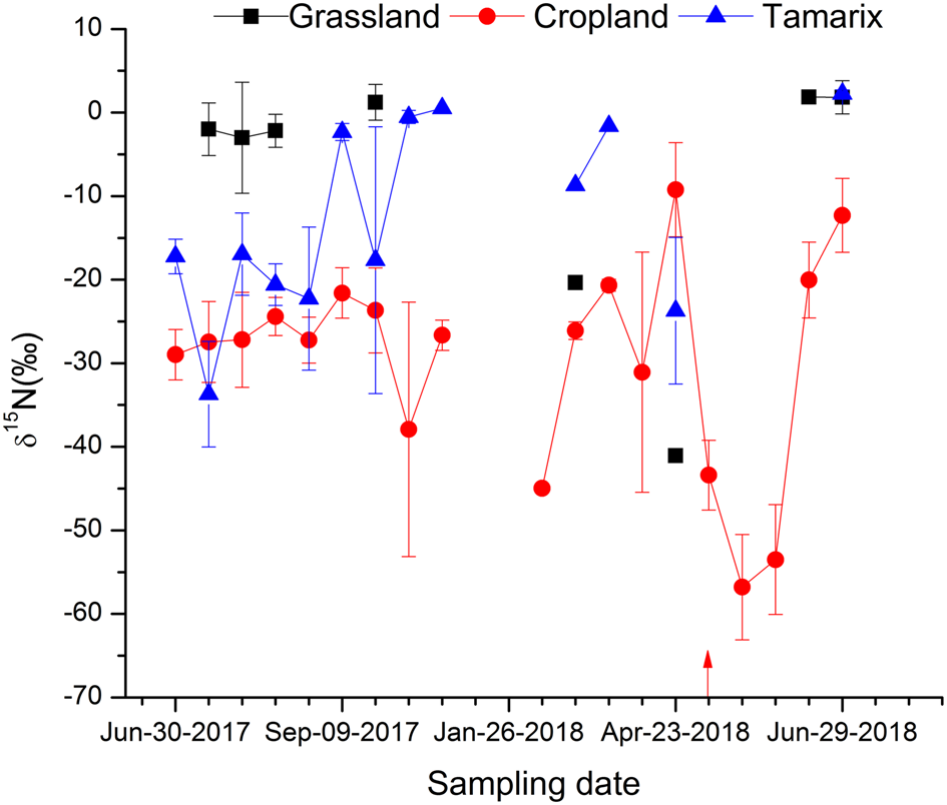
^15^N isotopic signature of soil-emitted N_2_O from studied ecosystems. Each point represents arithmetic mean of 1–4 replicates with standard errors. Arrow represents fertilizer application event in the cropland.

There was a significant difference (p < 0.01) in the ^15^N isotopic signature of soil-emitted N_2_O between the three ecosystems (Fig. 6). The difference between grassland and Tamarix was at the level of p < 0.01 while between grassland and cropland was at the level of p < 0.001, suggesting N addition has strong effect on depletion of ^15^N in N_2_O. N_2_O emitted from cropland was more depleted in ^15^N while N_2_O emitted from grassland was less depleted. The median ^15^N values in emitted N_2_O were − 0.18 ‰ (ranging from − 41.0 to 5.8‰, n = 14), − 25.3 ‰ (ranging from − 68.3 to 4.6 ‰, n = 63) and − 13.7 ‰ (ranging from − 50.5 to 3.0‰, n = 32) for the grassland, cropland and Tamarix, respectively. Due to problems with the IRMS, results from the beginning of the experiment are not included. In the grassland, due to low and negative fluxes of N_2_O, it was not always possible to calculate ^15^N values in soil-emitted N_2_O. Emitted N_2_O was more depleted in ^15^N in April in the grassland while in the Tamarix it was during the pill-bug decomposition period. In the cropland, it was just after the application of N fertilizer and this continued for about three weeks after the fertilization, then in the fourth week, when N_2_O emission reached its highest peak, the values returned to the normal range (Figs. 5, 6). There was no significant relationship between measured parameters and ^15^N in soil-emitted N_2_O.

## Discussion

At our experimental site, we had a unique opportunity to investigate the impact of land-use change (LUC) from natural to semi-natural and artificial ecosystems on N_2_O flux and its ^15^N within the same climatic conditions and soil type. LUC is associated with changes in various land cover types as a result of different management practices, which then can lead to changes in soil physical, chemical^41^ and biological properties^20^. The changes in these soil properties can alter soil greenhouse gas emissions^16,19^. Soil humidity, temperature, NH_4_ content and NO_3_ content are the major soil parameters that influence N_2_O emission from soil^21,36,42^. With the change in the land use, it was observed that these soil parameters were significantly influenced at our study site, which may have led to the differences in N_2_O flux from the different ecosystems.

In the grassland, no studied soil parameters were significantly correlated to N_2_O flux, which may have been due to limited NO_3_ content. The relatively high NH_4_ content and low NO_3_ in grassland soil indicates inhibition of nitrification process, causing low N_2_O emissions. The positive correlation between soil temperature and N_2_O emission in the cropland and Tamarix, observed in our study is consistent with other studies^36,43^ and can be explained by the increase in microbial activity with an increase in temperature^44^. WFPS higher than 80% is favorable for N_2_O reduction to N_2_^22^. Low N content along with higher WFPS and frequent N_2_O uptake results reported in the grassland site indicate that denitrification is a dominant process of N_2_O emission. Optimum WFPS for N_2_O emissions ranges from 60 to 80%^22^, and there have been reports of significant positive to negative or no relationship between WFPS and N_2_O emission^45–47^. Increase in soil moisture has a greater effect when dry soil is wetted^48^. So, higher WFPS (around 90%) in grassland and cropland might not be limiting factor controlling N_2_O emissions in our study. We only observed significant relationship between WFPS and N_2_O flux in Tamarix. The negative relationship might be due to excessive WFPS than that is required for optimum N_2_O production^49^. NH_4_ and NO_3_ are the main substrates for nitrification and denitrification^50,51^. Significant positive relationships between N_2_O emission and both NH_4_ and NO_3_ have previously been demonstrated^42^ indicating that coupled nitrification–denitrification contributes to N_2_O formation in the soil^50^. Similarly, in the current study positive relationships were found between N_2_O flux and NH_4_ and NO_3_ content in the cropland; however, only with NO_3_ in the Tamarix. It can be difficult to identify the N_2_O formation process responsible or the emissions i.e. either nitrification or denitrification, as both processes can occur simultaneously in the soil^50^. The results showing a range of both positive and negative relationships between various soil environmental parameters and N_2_O flux indicate that N_2_O formation processes have complex interactions with these soil parameters.

Often ecosystems with low N content have a negative flux and low annual N_2_O emission. The grassland site in our study was like most natural ecosystems^21,53^, N limited with low atmospheric nitrogen input and densely rooted vegetation and therefore emitted less N_2_O^54^. High WFPS with low N content favors denitrification leading to N_2_O consumption^53,55^. However, relatively dry ecosystems have also been reported to consume atmospheric N_2_O^56–58^; however, the possible mechanisms of N_2_O consumption by soil under dry conditions are not well understood^59^. N_2_O uptake has been observed at low NO_3_ levels (~ 1 mg N kg^−1^) and NH_4_ content (< 2 mg N kg^−1^) levels and high WFPS (90%)^60,61^. The grassland conditions in the current study were similar to these previous findings that may be the reason for N_2_O uptake occurring in the grassland in some sampling occasions. It has also been observed that soil under different plant species can have different rates of N_2_O reduction^62^ and that N_2_O consumption rate decreases with increase in soil NO_3_^63^. In the cropland and Tamarix systems in the present study, NO_3_ content was significantly higher than in the grassland, which might have resulted in a decrease in the reduction of N_2_O to N_2_, leading to the higher emission of N_2_O. The more depleted ^15^N values in soil-emitted N_2_O in the cropland and Tamarix compared to the grassland (Fig. 6) is further evidence of a decrease in the reduction of N_2_O to N_2_ in those systems^29,30^.

Overall, N_2_O flux in the grassland was low (4.0 N_2_O-N µg m^−2^ h^−1^) with an annual cumulative emission of 0.5 kg N_2_O-N ha^−1^ year^−1^. These findings are similar to those observed in other studies on natural grassland under different climatic conditions on non-saline soils^54,59,64–66^. However, compared to a saline grassland with the same dominant vegetation^36^ the flux rate in the current study was low. This was possibly due to the low NO_3_ and NH_4_ content. When natural grasslands with low N content are converted to cropland, the addition of a large amount of N fertilizer may potentially contribute to high N_2_O emissions^65^. Consistent with this, the cropland in the current study emitted about 7 times more N_2_O than the grassland. The annual N_2_O emission rate was similar to the IPCC default emission factor, i.e. 1% of applied N fertilizer is emitted as N_2_O in the agricultural fields^67^. The observed N_2_O emission from our cropland was lower than that from non-saline–alkaline soils in the same climatic area under application of the same amount of fertilizer^68^. Similarly, the N_2_O flux from some non-saline–alkaline soils, receiving a similar rate of fertilizer, was three times higher than from the cropland in our study^43^. A saline–alkaline sunflower field, receiving 300 kg N ha^−1^ year^−1^, emitted 9.8 kg N ha^−1^ year^−1^^10^, which is 3.8 times higher than the emission rate from the cropland in the current study, which had 400 kg N ha^−1^ year^−1^ applied. The Tamarix ecosystem emitted 2.6 times more N_2_O than the native grassland. This increase can be attributed to the higher NO_3_ content. The increase in NO_3_ content could also be linked to a lower reduction of N_2_O to N_2_ in the Tamarix system because high NO_3_ inhibits N_2_O reduction^69^. Conversion of grassland to tree plantations has a contrasting (increased to no influence) effect on N_2_O emission^17,18^. Overall, our results support our hypothesis that conversion of native grassland to cropland or Tamarix ecosystems would lead to change in soil environmental variables and an increase in N_2_O emission.

When compared with studies involving similar land use or land-use change (Supplement Information S1) our results from the respective ecosystems are within the ranges reported in the literature. This result suggests that saline–alkaline soils may not always have a higher potential for N_2_O emission, as hypothesized by Ghosh et al.^70^ and Yang et al.^10^. For the cropland the fertilizer application rate was higher than other studies in the literature (Supplement Information S1), this is likely to have led to the higher rate of N_2_O emission from the cropland. In saline–alkaline soil, NH_4_ can be converted to NH_3_ and lost to the atmosphere, which may decrease the probability of N_2_O formation due to nitrification^13^. Two meta-analyses^11,12^ reported that alkaline soils emit less N_2_O compared to natural and acidic soils. Furthermore, high salinity inhibits both nitrification and denitrification processes^8,9^. These negative effects of both salinity and alkalinity on N_2_O production processes and emissions further suggest that saline–alkaline soil may not emit more N_2_O.

It is evident from previous research^25,28^ that there may be differences in the ^15^N in soil-emitted N_2_O between fertilized and unfertilized ecosystems. Therefore, significant differences were expected in the ^15^N isotopic signatures in soil-emitted N_2_O between the unfertilized ecosystems (grassland and Tamarix) and the fertilized cropland. As there was no anthropogenic N input in grassland and Tamarix, our expectation was ^15^N in N_2_O would be similar in these two ecosystems. However, differences were observed among all three ecosystems. The ^15^N in N_2_O emitted from the grassland, cropland, and Tamarix were all within the range reported by other studies^25,26,28,62,71^. As we can see from Fig. 6 that temporal variability of ^15^N in soil-emitted N_2_O was highest in cropland, indicating that N cycling process in the cropland is relatively open. The more depleted ^15^N in N_2_O emitted from the cropland implies that N availability can be considered enhanced (due to the high rate of N fertilizer) in the ecosystem^25^. When nitrogen availability is enhanced, the N_2_O production process favors larger ^15^N fractionation, leading to more depleted ^15^N in N_2_O from the soil^25,72^. This phenomenon can lead to difference in the ^15^N in N_2_O emitted from the cropland compared to the grassland and Tamarix, as observed in this study. After application of fertilizer the cropland could be considered to have unlimited N availability so the N_2_O emitted was strongly depleted in ^15^N, indicating the production of N_2_O, either by nitrification or denitrification, favored larger ^15^N fractionation rather than shift from denitrification to nitrification^25,28,71,72^. Although ^15^N values in soil-emitted N_2_O can sometimes be used to predict sources of N_2_O when combined measurements of ^15^N values in substrates for N_2_O production^28^ and molecular analysis of N_2_O producing organisms^40^, with data from this trial was not possible to estimate relative contributions of nitrification and denitrification. Moreover, more powerful tools like ^15^N site preference (SP) is a good indicator of production pathways^24,73,74^, which was not used in this study, making difficult to generalize dominant process of N_2_O production in different ecosystems.

Contrarily to our hypothesis, there was a difference between ^15^N in soil-emitted N_2_O within unfertilized (grassland and tamarix) ecosystems. The reason for differences in the ^15^N in N_2_O between the grassland and Tamarix may be a difference in N_2_O reduction capability. It is likely that N_2_O reduction in the grassland (as evidenced by N_2_O consumption) enriched the ^15^N in N_2_O, so when it was emitted to the atmosphere it was less depleted than N_2_O emitted from soil in which reduction has not occured^29,75^. A possible reason for the reduction of N_2_O being favored in the grassland soil may be the low concentrations of NO_3_^69^ and high WFPS^22^. For this reason reduction of N_2_O to N_2_ might be more prominent in the grassland compared to the Tamarix. However, it could be a possibility that gross N_2_O consumption may be masked by higher rates of N_2_O production^76^ in the cropland and Tamarix. The ^15^N isotope content of the substrates (NH_4_ and NO_3_) for N_2_O production were not measured in the current study, which could have provided more insight into the reason for the observed differences between the ecosystems. The ^15^N differences in the emitted N_2_O between ecosystems could also be due to variation in the microbial community composition in the soils^77^. Several factors favor complete denitrification, such as differences in microbial community composition (denitrifiers), presence of denitrification enzymes, high soil water content, high soil pH, a low rate of O_2_ diffusion and presence of labile carbon^55^. So differences in those factors should not be ruled out as causes for the differences in the ^15^N content in emitted N_2_O between the ecosystems.

The ^15^N content in atmospheric N_2_O has been decreasing since the preindustrial age^78^; however, atmospheric N_2_O concentration is increasing^5^. This decrease in the ^15^N in N_2_O has been considered to be a result of an increase in the use of chemical fertilizer^5,28^. Moreover, global decline in the N_2_O reduction process relative to production might also contribute to the decrease in the ^15^N^29^. Our results indicate that the conversion of natural ecosystems to cropland with the addition of anthropogenic N would greatly contribute to the depletion of the ^15^N in atmospheric N_2_O by emitting more depleted ^15^N in N_2_O along with higher N_2_O emission rate, which was according to our hypothesis. Moreover, if ecosystems with more reduction capability (such as grassland) are converted to Tamarix that have less reduction capability (assumed due to the absence of measured atmospheric N_2_O consumption in our study), this would also play a role in the depletion of ^15^N in atmospheric N_2_O. Overall, it can be concluded that the addition of anthropogenic N to cropland would contribute more to deplete ^15^N in atmospheric N_2_O than any other processes.

## Conclusions

Our study showed that LUC from native grassland to Tamarix and cropland on saline–alkaline soil significantly influence soil temperature, soil moisture and NH_4_ and NO_3_ contents. The changes in these soil factors, along with the observed correlations between N_2_O fluxes and the soil parameters, could explain the differences in N_2_O flux caused by the LUC. Saline–alkaline soil may not always act as a potentially high source of N_2_O, as our fluxes and annual emissions result are in the usual ranges for the respective ecosystems reported in the literature. The conversion from native grassland to Tamarix ecosystem increased more N_2_O 2.6 times while cropland increased 7 times. The LUC also influenced the ^15^N in soil-emitted N_2_O, greatly depleting it in cropland and moderate in Tamarix compared to native grassland. The differences in the ^15^N in soil-emitted N_2_O between the fertilized and unfertilized ecosystems could be attributable to anthropogenic N fertilization. The differences in the ^15^N in N_2_O between the unfertilized ecosystems (grassland and Tamarix) could be attributable to the N_2_O reduction capacity of native grassland. Our results further suggest that the depletion of the ^15^N in atmospheric N_2_O since the pre-industrial age could be highly attributable to anthropogenic N addition and to lesser extent to land-use changes where ecosystems with more N_2_O reduction capacity have been converted to ecosystems with less N_2_O reduction capacity.

## Supplementary information

Supplementary Information.

## Data Availability

The datasets of the current study will be available from the corresponding author on reasonable request.
