# Supplementary material for: Nitrogen isotopic signatures and fluxes of N2O in response to land-use change on naturally occurring saline–alkaline soil
Source: Sci Rep. 2020 Dec 4;10:21253. doi: 10.1038/s41598-020-78149-w (PMC7718238; doi:10.1038/s41598-020-78149-w)
Supplement: Supplementary file 1 — Supplementary Information. [file 41598_2020_78149_MOESM1_ESM.docx]

**Nitrogen isotopic signatures and fluxes of N_2_O in response to land-use change on naturally occurring saline-alkaline soil**

Arbindra Timilsina^1,2,4,*^, Wenxu Dong^1,4^, Jiafa Luo^3^, Stuart Lindsey^3^, Yuying Wang^1^, Chunsheng Hu ^1,2,*^

^1^ Key Laboratory of Agricultural Water Resources, Center for Agricultural Resources Research, Institute of Genetics and Developmental Biology, Chinese Academy of Sciences, Shijiazhuang 050021, China

^2^ University of Chinese Academy of Sciences, Beijing 100049, China

^3^ Land and Environment, AgResearch, Hamilton 3240, New Zealand

^4^ These authors contributed equally: Arbindra Timilsina and Wenxu Dong.

**Supplement 1**

**Table 1**: Compilation of annual N_2_O emission rate (kg N_2_O-N ha^-1^ yr^-1^) for studies with similar land use or LUC to the current study. (n.a denotes not available).

| S.N | Annual N_2_O emission | pH (soil depth) | Comments | References |
| --- | --- | --- | --- | --- |
| 1. | 0.73 | 6.9 (0-20 cm) | Grassland, *Leymus chinensis*, negative flux reported during winter only | ^1^ |
| 2. | 0.37 | 7.35 (0-20 cm) | Grassland, *Kobresia humilis*, *Festuca ovina*, *Elymus*  *nutans*, *Poa pratensis* | ^2^ |
| 3. | 0.3  0.6  2.0  1.4  3.8 | 5.8 (0-25 cm)  5.3 (0-19 cm)  5.5 (0-26 cm)  5.3 (0-13 cm)  6.1 (0-15 cm) | Grassland: *Phleum pretense* L.  *Phalaris arundinacea* and *Aleopecurus pratensis* L.  *Dactylis glomerata* L. and *Festuca paratensis*  *Dactylis glomerata* L. and *Lolium multiflorum* Lam.  *Lolium multiforim* lam. And *Digitaria ciliaris* Retz. | ^3^ |
| 4. | 0.77  0.9  0.83 | n.a | Grassland: *Festuca pseudovina*, *Carex stenophylla*, *Cynodon dactylon*  *Dactylis glomerata* and *Salvia nemorosa*  *Dactylis glomerata* and *Salvia nemorosa*  *Festuca rupicola* and *D. glomerata* | ^4^ |
| 5. | 0.16 | 6.5 (0-15 cm) | Grassland: *Bouteloua gracilis* | ^5^ |
| 6. | 0.22 | 8.6 (0-10 cm) | Grassland: *Stipa breviflora*, *Artemisia frigida*, *Cleistogenes songorica* | ^6^ |
| 7. | 0.5 | 8.74 (0-20 cm) | Grassland: *Phragmites australis* | This study |
| 8. | 0.94 | 7.9 (0-20cm) | 100 kg N ha^-1^yr^-1^, Cotton filed | ^7^ |
| 9. | 2.6 | 8.0 (0-10 cm) | 66.3 kg N ha^-1^yr^-1^ in the year of the experiment while before the year was double, cotton field | ^8^ |
| 10. | 1.69 | 7.2 (0-10 cm) | 200 kg N ha^-1^yr^-1^,  Measurement period 7 months,  cotton field | ^9^ |
| 11. | 0.59  1.94 | 7.3 (0-10 cm) | No fertilizer  270 kg N ha^-1^yr^-1^,  cotton field | ^10^ |
| 12. | 1.43 | 8.7 (0-10 cm) | 75 kg N ha^-1^yr^-1^ | ^11^ |
| 13. | 0.55  0.67  1.07  1.89 | 7.3 | No fertilizer  90 kg N ha^-1^yr^-1^  180 kg N ha^-1^yr^-1^  270 kg N ha^-1^yr^-1^,  cotton field | ^12^ |
| 14. | 1.3 | n.a | 177 kg N ha^-1^yr^-1^,  cotton field | ^13^ |
| 15. | 3.5 | 8.45 | 400 kg N ha^-1^yr^-1^,  cotton field | This study |
| 16. | 10.12  0.07  0.77 | 4.36 (0-10 cm)  7.22  7.19 | Grassland converted Sitka spruce (Picea sitchensis) forest before 16 years ago. (the control site grassland emitted 0.12 kg N ha^−1^ yr^−1^)  Grassland converted to forest 6 years old Fraxinus excelsior  Grassland converted to forest 12 years old Fraxinus excelsior | ^14^ |
| 17. | 3.38 | 4.5 (0-20 cm) | Grassland converted to *Pinus elliottii* Engelm plantation before 29 years ago | ^15^ |
| 18. | 0.24 | n.a | 6 years Acacia plantation converted from grassland (grassland emitted 0.31 kg N ha-1 yr-1) | ^16^ |
| 19. | 0.9 | 5.5 | 20-year-old Secondary forest regenerated from grassland (Vegetation not specified) | ^17^ |
| 20. | 1.3 | 8.58 | Converted from native grassland to *Tamarix chinensis* | This study |

**References:**

1. Du, R., Lu, D. & Wang, G. Diurnal, seasonal, and inter-annual variations of N_2_O fluxes from native semi-arid grassland soils of inner Mongolia. *Soil Biol. Biochem.* **38**, 3474–3482 (2006).

2. Hu, Y. *et al.* Effects of warming and grazing on N_2_O fluxes in an alpine meadow ecosystem on the Tibetan plateau. *Soil Biol. Biochem.* **42**, 944–952 (2010).

3. Shimizu, M. *et al.* The effect of fertilizer and manure application on CH_4_ and N_2_O emissions from managed grasslands in Japan. *Soil Sci. Plant Nutr.* **59**, 69–86 (2013).

4. Horváth, L. *et al.* Estimation of nitrous oxide emission from Hungarian semi-arid sandy and loess grasslands; effect of soil parameters, grazing, irrigation and use of fertilizer. *Agric. Ecosyst. Environ.* **139**, 255–263 (2010).

5. Mosier, A. R. *et al.* CH_4_ and N_2_O fluxes in the Colorado shortgrass steppe: 2. Long‐term impact of land use change. *Global Biogeochem. Cycles* **11**, 29–42 (1997).

6. Wang, Z. *et al.* Influence of increasing temperature and nitrogen input on greenhouse gas emissions from a desert steppe soil in Inner Mongolia. *Soil Sci. Plant Nutr.* **57**, 508–518 (2011).

7. Mahmood, T., Ali, R., Iqbal, J. & Robab, U. Nitrous oxide emission from an irrigated cotton field under semiarid subtropical conditions. *Biol. Fertil. Soils* **44**, 773–781 (2008).

8. Liu, C. *et al.* Nitrous oxide and nitric oxide emissions from an irrigated cotton field in Northern China. *Plant Soil* **332**, 123–134 (2010).

9. Scheer, C., Grace, P. R., Rowlings, D. W. & Payero, J. Soil N_2_O and CO_2_ emissions from cotton in Australia under varying irrigation management. *Nutr. Cycl. Agroecosystems* **95**, 43–56 (2013).

10. Grace, P., Shcherbak, I., Macdonald, B., Scheer, C. & Rowlings, D. Emission factors for estimating fertiliser-induced nitrous oxide emissions from clay soils in Australia’s irrigated cotton industry. *Soil Res.* **54**, 598 (2016).

11. Wang, K. *et al.* Comparison between static chamber and tunable diode laser-based eddy covariance techniques for measuring nitrous oxide fluxes from a cotton field. *Agric. For. Meteorol.* **171**–**172**, 9–19 (2013).

12. Scheer, C., Rowlings, D. W. & Grace, P. R. Non-linear response of soil N_2_O emissions to nitrogen fertiliser in a cotton–fallow rotation in sub-tropical Australia. *Soil Res.* **54**, 494 (2016).

13. Macdonald, B. C. ., Chang, Y. F. & Warneke, S. Potential contributions of surface and ground water to nitrous oxide emissions from irrigated cotton production systems. *Agric. Water Manag.* **168**, 78–84 (2016).

14. Benanti, G., Saunders, M., Tobin, B. & Osborne, B. Contrasting impacts of afforestation on nitrous oxide and methane emissions. *Agric. For. Meteorol.* **198**–**199**, 82–93 (2014).

15. Wang, Y. *et al.* Effect of litter layer on soil–atmosphere N_2_O flux of a subtropical pine plantation in China. *Atmos. Environ.* **82**, 106–112 (2014).

16. de Godoi, S. G. *et al.* The conversion of grassland to acacia forest as an effective option for net reduction in greenhouse gas emissions. *J. Environ. Manage.* **169**, 91–102 (2016).

17. Verchot, L. V. *et al.* Land use change and biogeochemical controls of nitrogen oxide emissions from soils in eastern Amazonia. *Global Biogeochem. Cycles* **13**, 31–46 (1999).
